# Stress and feeding choices: How do socio-demographic factors shape formula use among Polish mothers?

**DOI:** 10.3389/fpubh.2025.1697153

**Published:** 2025-12-01

**Authors:** Agnieszka Czerwińska-Osipiak, Anna Weronika Szablewska, Krzysztof Jurek, Wiktoria Karasek, Conceição Santiago

**Affiliations:** 1 Department of Obstetric and Gynaecological Nursing, Institute of Nursing and Midwifery, Faculty of Health Sciences with the Institute of Maritime and Tropical Medicine, Medical University of Gdańsk, Gdańsk, Poland; 2 Institute of Sociological Sciences, Faculty of Social Sciences, John Paul II Catholic University of Lublin, Lublin, Poland; 3 Higher School of Health, Santarém Polytechnic University, Santarém, Portugal; 4 Research and Innovation in Health—RISE-Health, Faculty of Medicine, University of Porto, Porto, Portugal

**Keywords:** breastfeeding, infant formula, maternal stress, socioeconomic factors, feeding behavior

## Abstract

**Background:**

Breastfeeding is widely recognized as the gold standard in infant nutrition, yet many women discontinue exclusive breastfeeding earlier than recommended. Maternal stress has been identified as a potential factor contributing to breastfeeding difficulties; however, its interaction with socio-demographic variables remains insufficiently explored. In Poland, where breastfeeding initiation rates are high but exclusive breastfeeding drops to only 4% by six months postpartum, understanding these interactions is essential to improve breastfeeding outcomes.

**Objective:**

In this study, it was examined whether maternal stress moderates the relationship between socio-demographic factors and the introduction of formula supplementation among Polish mothers.

**Methods:**

An observational, cross-sectional study was conducted among 1,092 mothers of infants ≤6 months. Data were collected via an anonymous online questionnaire assessing socio-demographic characteristics, feeding practices and maternal stress using the Perceived Stress Scale (PSS-10). Logistic regression models were applied separately for low/moderate and high stress groups.

**Results:**

Socio-demographic influences on formula supplementation were moderated by maternal stress level. In high-stress mothers, older age (AOR ≈ 1.81, *p* = 0.026) was associated with higher odds of supplementation, whereas parity (AOR ≈ 0.52, *p* = 0.030) and better housing conditions (AOR ≈ 0.50, *p* = 0.010) were connected with lower odds (Nagelkerke R^2^ ≈ 0.084). In the low/moderate stress group, the model was not significant.

**Conclusion:**

Maternal stress plays a moderating role in the relationship between socio-demographic variables and infant feeding practices. These findings provide novel evidence that psychological and environmental factors jointly shape feeding decisions. Integrating stress assessment and psychosocial support into postnatal and breastfeeding care may strengthen public health interventions aimed at sustaining exclusive breastfeeding in Poland.

## 1 Introduction

Contemporary motherhood, while often idealized, represents a period of profound transformation and substantial challenges that entail a considerable risk of chronic stress. The adverse consequences of such stress extend well beyond psychological discomfort, contributing to the onset of mood disorders, diminished quality of mother-infant interactions and long-term health complications affecting both the mother and the child (1, 2). The postpartum period, marked by physiological instability, caregiving demands and identity reorganization, is considered critical for a woman’s psychological well-being (3).

In this context, decisions regarding infant feeding occupy a central place in the mother’s experience, carrying a substantial emotional and social burden. Despite the global recommendations of the World Health Organization (WHO) advocating exclusive breastfeeding during the first six months of life, a considerable proportion of mothers introduce formula milk into the infant’s diet, either as a supplement (mixed feeding) or as a complete replacement for breastfeeding (4, 5).

Poland stands out with a relatively high share of women initiating breastfeeding compared to other European countries and the world (6, 7). European breastfeeding rates show considerable variation, with exclusive breastfeeding at six months ranging from 13 to 39% (8, 9), falling short of the WHO’s 50% target (10) and indicating persistent multi-level barriers to breastfeeding continuation (11–13). This highlights the need for national governments to implement consistent, systemic strategies for monitoring and promoting breastfeeding with financial and political support (10).

Breastfeeding practices are shaped by a complex interplay of factors including sociodemographic determinants, cultural norms and individual behaviors (8). Psychosocially, we apply the Conservation of Resources (COR) theory, which posits that individuals strive to retain and protect their resources (e.g., energy, time, emotional capacity) (14). High stress depletes these vital resources, making it more difficult to engage in demanding behaviors such as exclusive breastfeeding. In this context, socio-demographic factors (e.g., housing conditions, age, parity) can act as either additional stressors that further deplete resources or as protective buffers that help conserve them. Within this framework, we ask: Does stress moderate socio-demographic influences on formula supplementation? Which factors operate under low/moderate versus high stress? We hypothesize high stress amplifies socio-demographic influences as depleted resources increase vulnerability to circumstances. However, the potential moderating role of stress in this relationship remains poorly understood, particularly in the Polish context, which presents a striking paradox: despite remarkably high initiation rates (97%), exclusive breastfeeding rates decline dramatically to just 4% by six months postpartum (6–8, 11). This sharp decline represents one of Europe’s most significant drops in exclusive breastfeeding rates, suggesting the presence of country-specific moderating factors that may influence how stress affects feeding decisions. Despite extensive international literature, comprehensive research focused specifically on the Polish population remains limited. Current data, primarily from the Central Statistical Office (GUS) surveys, including the European Health Interview Survey (EHIS) from 2014 and 2019, offer insights into early breastfeeding but lack depth on duration and practices (15). This is the first large-scale study in Poland to investigate the moderating role of maternal stress in the relationship between socio-demographic factors and infant feeding practices. By examining stress as a moderating variable rather than merely a predictor, this study provides novel insights into the complex mechanisms underlying feeding decisions. The research is guided by a conceptual framework that integrates physiological and psychosocial pathways to explain how maternal stress moderates socio-demographic effects.

## 2 Materials and methods

### 2.1 Study design and participants

This observational, cross-sectional study was conducted among Polish mothers in the postpartum period (up to six months after delivery). Data were collected via an anonymous online questionnaire completed by the participants themselves. The survey covered socio-demographic characteristics, maternal stress levels and infant feeding practices. Stress was assessed using the validated Polish version of the Perceived Stress Scale (PSS-10), and women were categorized into low, moderate and high stress groups.

Prior to the analyses, missing data were examined for all variables included in the study. The pattern of missingness was assessed to determine whether data were missing completely at random, at random or not at random. For subsequent analyses, listwise deletion was applied, whereby cases with missing values for any of the variables included in a given model were excluded. This approach pre-serves the integrity of parameter estimates and minimizes the risk of introducing significant bias, particularly when the proportion of missing data is low.

A total of 1,092 women were included in the final sample. Participants were recruited from online platforms and social media groups dedicated to motherhood from across Poland. This nationwide study was coordinated by researchers from the Medical University of Gdańsk, ensuring a standardized approach to data collection. To minimize selection bias, private and local groups were excluded, as were groups specifically focused on breastfeeding, to ensure the inclusion of participants with varying feeding preferences and experiences. Inclusion criteria required the child’s age to be ≤6 months; inconsistent or contradictory responses were excluded. A flow chart illustrating the number of respondents at each stage and the application of the inclusion/exclusion criteria is available in the Supplementary Figure S1. All participants provided informed consent. Ethical approval was obtained from the Bioethics Commission of the Medical University of Gdańsk (NKBBN/159/2023). All procedures followed the principles of the 1964 Declaration of Helsinki.

### 2.2 Data collection

The questionnaire was created by a team of certified lactation consultants and included demographic as well as socioeconomic variables (age, education, marital status, place of residence, housing conditions, income, employment and parity) and infant feeding practices (exclusive breastfeeding, mixed feeding, formula feeding and timing of supplementation). Maternal stress was measured using the PSS-10. Cronbach’s alpha for the scale was 0.90.

### 2.3 Variables

The perceived stress variable was categorized into two groups for analysis: women with low/moderate stress and women with high stress. Based on the PSS-10 scale scoring guidelines, scores of 0–13 were classified as low, 14–19 as moderate, and 20–40 as high perceived stress levels. The overall mean score on the PSS-10 scale was 19.17 (SD = 7.05; Me = 19.00), with values ranging from 0 to 39. Analysis of the score distribution across three stress intensity categories indicated that participants with low stress scored, on average, 9.75 points (SD = 3.10; Me = 11.00), ranging from 0 to 13 points. In the moderate stress group, the mean score was 16.55 (SD = 1.61; Me = 17.00; range 14–19), whereas participants with high stress achieved a mean score of 25.27 (SD = 4.18; Me = 25.00; range 20–39). For analytical purposes, the low and moderate categories were combined into a single low/moderate stress group, which was compared with the high stress group.

The combination of low and moderate stress categories is justified, at the same time, theoretically and practically. Both levels fall within the range of adaptive responses to stressors and do not lead to a disruption of individual functioning. According to the transactional model of stress by R. S. Lazarus and S. Folkman, it results from the cognitive appraisal of a situation in the context of available resources and coping abilities. At low and moderate levels, stress may serve a mobilizing function (eustress), promoting resource activation and effective action. In contrast, high stress (distress) is associated with exceeding adaptive capacities and the emergence of negative psychological or somatic consequences.

The sociodemographic variables were included as predictors and coded as binary (0/1). These variables were: number of children, place of residence (large city vs. village/small town), level of education (higher vs. primary/vocational/secondary), professional activity (no/yes), marital status (single vs. married or in partner-ship), assessment of financial situation (good/sufficient vs. very good), assessment of housing conditions (good/sufficient vs. very good), and age (reference category vs. below 30 years). For each variable, one category was treated as the reference (coded 0), while the comparative category was coded as 1. This dummy coding allowed the inclusion of qualitative characteristics in the statistical models.

Throughout the manuscript, “formula supplementation” denotes any formula use alongside breastfeeding (mixed feeding), whereas “formula feeding” denotes exclusive use of formula.

### 2.4 Statistical analysis

Data were analyzed using IBM SPSS Statistics. Logistic regression models were initially applied to examine the associations between socio-demographic variables and formula supplementation across stress-level groups. Prior to conducting the logistic regression analysis, collinearity diagnostics were performed to assess potential multicollinearity among independent variables. Variance Inflation Factor (VIF) values were calculated using a linear regression model including the same predictors, and all values were below 5, indicating no evidence of problematic collinearity.

Logistic regression results were reported as regression coefficients (B), standard errors (SE), Wald χ^2^ values, *p*-values, and odds ratios (OR) with 95% confidence intervals (CI). Model fit was assessed using the Hosmer–Lemeshow goodness-of-fit test, and explanatory power was evaluated with Cox & Snell and Nagelkerke R^2^ coefficients. The omnibus test of model coefficients was used to assess overall model significance.

Binary logistic regression is the most commonly used traditional method for dichotomous outcomes; however, it also has some limitations. Because the outcome variable (formula supplementation) had a prevalence greater than 10%, odds ratios could overestimate the true relative risk. Therefore, multilevel Poisson regression with robust variance was also applied in both bivariate and multivariate analyses to directly estimate prevalence ratios (PRs) and their 95% confidence intervals, providing a more accurate measure of association for common outcomes while maintaining comparability with conventional logistic regression results.

## 3 Results

### 3.1 Study group characteristics

Of the 1,092 postpartum women included in the study, 1,026 (94.0%) initiated breastfeeding, whereas 66 (6.0%) did not attempt breastfeeding due to personal choices or clinical circumstances. Among all participants, 21% (*n* = 229) reported supplementing their infants with formula. The prevalence of formula supplementation was 19.2% in the low/moderate stress group and 23.6% in the high stress group. The mean age was 30.63 years (SD = 4.04), ranging from 18 to 44 years. Detailed demographic and socio-economic characteristics of the study population are presented in Table 1.

**Table 1:** Study group characteristics.

Characteristics	No.	%
Respondents	1,092	100
Age		
<25	102	9.3
26–35	848	77.7
36–45	142	13
Education level		
Primary education	1	0.1
Secondary education	172	15.8
Higher education (bachelor’s/master’s degree)	900	82.4
Vocational education	19	1.7
Place of residence		
Village	252	23.1
City < 50 k residents	181	16.6
City 50–150 k residents	161	14.7
City 150–500 k residents	180	16.5
City > 500 k residents	318	29.1
Marital status		
Single	70	6.4
Married/Partnership	1,011	92.6
Divorced	11	1
Financial situation		
Very good	336	30.8
Good	528	48.4
Satisfactory	214	19.6
Bad	10	0.9
Very bad	4	0.4
Professional activity		
Yes	857	78.5
No	235	21.5
Housing conditions		
Very good	598	54.8
Good	424	38.8
Satisfactory	64	5.9
Bad	6	0.5
Very bad	0	0
Parity		
1	765	70.1
2	280	25.6
3	40	3.7
4	5	0.5
5 or more	2	0.2
Level of perceived stress		
Low	235	21.5
Moderate	412	37.7
High	445	40.8

### 3.2 Outcomes

Building on our previously published findings (16), we extended the analysis to examine the association between infant formula supplementation and socio-demographic characteristics, while accounting for maternal perceived stress levels.

The logistic regression models demonstrated acceptable fit (Hosmer–Lemeshow: low/moderate stress χ^2^ = 11.67, *p* = 0.166; high stress χ^2^ = 11.37, *p* = 0.182), with modest explanatory power (Nagelkerke R^2^ = 0.026 and 0.084 for low/moderate and high stress models, respectively). The omnibus test indicated that the model was not significant in the low/moderate stress group (χ^2^ = 10.47, *p* = 0.234) but significant in the high stress group (χ^2^ = 25.68, *p* = 0.001), underlining a moderation pattern by stress level.

In the high stress group, several socio-demographic factors emerged as significant predictors. For age, the crude analysis showed that mothers aged 30 years and older had higher odds of formula supplementation (crude OR = 2.222; 95% CI: 1.383–3.570; *p* < 0.001) and higher prevalence (crude PR = 1.863; 95% CI: 1.275–2.722; *p* = 0.001). In the adjusted model, these associations remained significant, with older mothers demonstrating higher odds (adjusted OR = 1.807; 95% CI: 1.072–3.047; *p* = 0.026) and higher prevalence (adjusted PR = 1.566; 95% CI: 1.025–2.392; *p* = 0.038). Housing conditions showed a consistent protective effect. Mothers living in very good housing had lower crude odds of formula supplementation (crude OR = 0.577; 95% CI: 0.368–0.905; *p* = 0.017) and lower prevalence (crude PR = 0.655; 95% CI: 0.462–0.929; *p* = 0.018). This association remained in the adjusted models (adjusted OR = 0.504; 95% CI: 0.298–0.851; *p* = 0.010; adjusted PR = 0.600; 95% CI: 0.409–0.881; *p* = 0.009). Parity was also a significant predictor. Multiparous mothers had lower odds in the crude analysis (crude OR = 0.420; 95% CI: 0.246–0.718; *p* = 0.002) and lower prevalence (crude PR = 0.502; 95% CI: 0.322–0.783; *p* = 0.002). These results persisted after adjustment (adjusted OR = 0.524; 95% CI: 0.292–0.940; *p* = 0.030; adjusted PR = 0.603; 95% CI: 0.367–0.990; *p* = 0.046).

In the high stress group, several socio-demographic factors emerged as significant predictors. For age, the crude analysis did not show a significant association with formula supplementation (OR = 0.959; 95% CI: 0.907–1.013; *p* = 0.131), whereas in the adjusted model, mothers aged 30 years and older had a significantly higher odds of formula supplementation (OR = 1.807; 95% CI: 1.072–3.047; *p* = 0.026) Housing conditions showed a consistent effect: very good housing was associated with a lower odds of formula supplementation in both the crude (OR = 0.577; 95% CI: 0.368–0.905; *p* = 0.017) and adjusted models (OR = 0.504; 95% CI: 0.298–0.851; *p* = 0.010). Parity also remained significant, with mothers having previous children demonstrating a lower odds of formula supplementation in both crude (OR = 0.420; 95% CI: 0.246–0.718; *p* = 0.002) and adjusted models (OR = 0.524; 95% CI: 0.292–0.940; *p* = 0.030). Other factors—including place of residence, education, professional activity, marital status and financial situation—were not significantly associated with formula supplementation in either the crude or adjusted models.

These results indicate that controlling for other variables can substantially change the direction and size of some effects, particularly for age and parity, highlighting the importance of adjusted models in behavioral research. The results suggest that maternal stress moderates the relationship between socio-demographic characteristics and formula use, amplifying the impact of experience- and resource-related factors in conditions of higher psychological strain.

This pattern indicates that under high stress, maternal experience and living conditions play a stronger role in shaping feeding decisions. Older or multiparous mothers may rely more on pragmatic coping strategies, such as formula supplementation, whereas better housing conditions likely reflecting greater socioeconomic stability may encourage continued breastfeeding through better access to resources and social support.

The results of detailed logistic regression are presented in Tables 2, 3.

**Table 2:** Crude odds ratios (OR) and crude prevalence ratios (PR) for socio-demographic predictors of formula supplementation, stratified by stress level (bivariate analysis).

Variable	Grouping of variable	Crude OR	95%CI	*p*	Crude PR	95%CI	*p*
Stress level—low/moderate							
Age	Below 30 years	1.000		0.401	1.000		0.401
	30 years and older	1.183	0.799–1.750		1.145	0.834–1.572	
Place of residence	Village/small town	1.000		0.532	1.000		0.553
	Large city	0.879	0.587–1.317		0.901	0.649–1.251	
Education	Higher	1.000		0.758	1.000		0.757
	Primary/vocational/secondary	1.081	0.657–1.779		1.065		
Professional activity	No	1.000		0.330	1.000		
	Yes	1.291	0.772–2.160		1.233	0.804–1.892	0.337
Marital status	Single	1.000		0.190	1.000		0.173
	Marriage/married or in a partnership	0.651	0.342–1.238		0.715	0.442–1.158	
Housing conditions	Good/sufficient	1.000		0.073	1.000		0.076
	Very good	1.458	0.965–2.203		1.360	0.969–1.910	
Financial situation	Good/sufficient	1.000		0.995	1.000		0.995
	Very good	1.001	0.662–1.514		1.001	0.717–1.398	
Parity	No	1.000		0.101	1.000		0.107
	Yes	0.680	0.429–1.078		0.728	0.496–1.070	
Stress level—high							
Age	Below 30 years	**1.000**		**<0.001**	**1.000**		**0.001**
	30 years and older	**2.222**	**1.383–3.570**		**1.863**	**1.275–2.722**	
Place of residence	Village/small town	1.000		0.532	1.000		0.332
	Large city	0.879	0.587–1.317		1.181	0.844–1.651	
Education	Higher	1.000		0.593	1.000		0.588
	Primary/vocational/secondary	1.170	0.658–2.081		1.226	0.733–1.729	
Professional activity	No	1.000		0.641	1.000		0.643
	Yes	1.133	0.670–1.915		1.101	0.733–1,655	
Marital status	Single	1.000		0.523	1.000		0.535
	Marriage/married or in a partnership	1.384	0.511–3.749		1.292	0.575–2.902	
Housing conditions	Good/sufficient	**1.000**		**0.017**	**1.000**		**0.018**
	Very good	**0.577**	**0.368–0.905**		**0.655**	**0.462–0.929**	
Financial situation	Good/sufficient	1.000		0.878	1.000		0.878
	Very good	0.962	0.583–1.585		0.970	0.661–1.424	
Parity	No	**1.000**		**0.002**	**1.000**		**0.002**
	Yes	**0.420**	**0.246–0.718**		**0.502**	**0.322–0.783**	

Crude ORs were obtained from univariate logistic regression; adjusted ORs were estimated from multivariate logistic regression models including all listed predictors. Crude PRs were obtained from univariate Poisson regression models with a log link; adjusted PRs were estimated from multivariate Poisson regression models including all listed predictors. Reference categories are indicated as 1. Statistically significant values (*p* < 0.05) are in bold. *N* = 1,092.

**Table 3:** Adjusted odds ratios (OR) and adjusted prevalence ratios (PR) with 95% confidence intervals for socio-demographic predictors of formula supplementation, stratified by stress level (multivariate analysis).

Variable	Grouping of variable	Adjusted OR	95%CI	*p*	Adjusted PR	95%CI	*p*
Stress level—low/moderate							
Age	Below 30 years	1.000		0.532	1.000		0.549
	30 years and older	1.143	0.752–1.736		1.111	0.788–1.566	
Place of residence	Village/small town	1.000		0.408	1.000		0.411
	Large city	0.839	0.553–1.272		0.869	0.823–1.610	
Education	Higher	1.000		0.584	1.000		0.580
	Primary/vocational/secondary	1.167	0.671–2.028		1.129	0.575–1.363	
Professional activity	No	1.000		0.263	1.000		0.248
	Yes	1.370	0.790–2.374		1.288	0.506–1.193	
Marital status	Single	1.000		0.302	1.000		0.292
	Marriage/married or in a partnership	0.703	0.360–1.373		0.764	0.793–2.162	
Housing conditions	Good/sufficient	**1.000**		**0.037**	**1.000**		**0.037**
	Very good	**1.634**	**1.030–2.592**		**1.481**	**0.467–0.976**	
Financial situation	Good/sufficient	1.000		0.346	1.000		0.297
	Very good	0.800	0.504–1.271		0.840	0.844–1.747	
Parity	No	1.000		0.183	1.000		0.199
	Yes	1.384	0.858–2.233		0.768	0.513–1.149	
Stress level—high							
Age	Below 30 years	**1.000**		**0.026**	**1.000**		**0.038**
	30 years and older	**1.807**	**1.072–3.047**		**1.566**	**1.025–2.392**	
Place of residence	Village/small town	1.000		0.368	1.000		0.340
	Large city	1.243	0.774–1.997		0.852	0.613–1.184	
Education	Higher	1.000		0.531	1.000		0.501
	Primary/vocational/secondary	1.222	0.653–2.289		1.156	0.758–1.764	
Professional activity	No	1.000		0.409	1.000		0.396
	Yes	1.271	0.719–2.247		1.187	0.799–1.762	
Marital status	Single	1.000		0.461	1.000		0.482
	Marriage/married or in a partnership	1.474	0.526–4.132		1.335	0.596–2.991	
Housing conditions	Good/sufficient	**1.000**		**0.010**	**1.000**		**0.009**
	Very good	**0.504**	**0.298–0.851**		**0.600**	**0.409–0.881**	
Financial situation	Good/sufficient	1.000		0.416	1.000		0.385
	Very good	1.275	0.710–2.287		1.205	0.792–1.834	
Parity	No	**1.000**		**0.030**	**1.000**		**0.046**
	Yes	**0.524**	**0.292–0.940**		**0.603**	**0.367–0.990**	

Crude ORs were obtained from univariate logistic regression; adjusted ORs were estimated from multivariate logistic regression models including all listed predictors. Crude PRs were obtained from univariate Poisson regression models with a log link; adjusted PRs were estimated from multivariate Poisson regression models including all listed predictors. Reference categories are indicated as 1. Statistically significant values (*p* < 0.05) are in bold. *N* = 1,092.

## 4 Discussion

In the present study, the relationship between stress level and socio-demographic predictors of formula supplementation was examined among women. Using logistic regression models, we found that only a limited number of demographic and socioeconomic factors were significantly associated with formula supplementation and that the pattern of predictors differed depending on the level of perceived stress.

While in previous studies it has been shown that attitudes towards breastfeeding have evolved alongside the growing availability of infant formula, resulting in persistently low rates of exclusive breastfeeding worldwide (17, 18), our findings highlight the role of maternal stress as an additional contextual factor shaping these decisions. Despite global and national efforts to promote exclusive breastfeeding, rates remain below WHO targets in most regions, including Poland (5, 8, 19, 20).

### 4.1 Biological and psychological mechanisms

Explaining the physiological mechanisms through which stress affects exclusive breastfeeding, it must be considered that psychological stress can disrupt the release of oxytocin—a hormone playing a key role in the milk ejection reflex during lactation. Continuous impairment of the milk ejection reflex can lead to decreased milk production due to incomplete breast emptying during each feeding. Maternal anxiety, as part of this physiological mechanism, causes an increase in serum cortisol levels and reduced insulin sensitivity, resulting in lowered milk production. This creates a vicious cycle: maternal anxiety, insufficient milk supply and inadequate infant weight gain, which can lead to the decision of introducing formula into an infant’s diet. However, the relationship between psychological stress and breastfeeding is bidirectional. The act of breastfeeding reduces maternal stress, likely through its influence on pleasure centers and the calming effect exerted by oxytocin on the mother. Researchers’ observations suggest that interventions supporting lactation and breastfeeding in women with high levels of psychological distress would benefit both maternal and child well-being, and implementing stress reduction programs could improve breastfeeding rates (13, 21).

### 4.2 Main findings and comparison with previous studies

The conducted analysis revealed that the varied nature of the relationships between socio-demographic characteristics and formula supplementation was not uniform but moderated by maternal stress levels. Beyond physiological mechanisms, a broader psychological context must be considered. In studies, it is shown that pregnancy and the postpartum period involve numerous psychosocial stressors that can negatively affect both maternal and infant health (22). Psychological factors, such as high stress levels, fatigue, self-efficacy and concerns about providing adequate infant nutrition, represent significant barriers to maintaining exclusive breastfeeding (21, 23). In a systematic review by Hoff et al. (24), maternal anxiety was identified as a major predictor of early breastfeeding cessation. These stressors—together with limited social support—create conditions that correlate with earlier cessation of exclusive breastfeeding. Current evidence further indicates that the co-occurrence of lactation difficulties and elevated stress levels are important warning signs that may precede the onset of postpartum depression (25, 26).

In addition to psychosocial stressors and postpartum depression, other maternal and contextual factors have been known to contribute to early breastfeeding cessation. Cesarean delivery, often associated with greater maternal stress and delayed lactation initiation, has consistently been linked to a higher risk of discontinuing exclusive breastfeeding compared to vaginal delivery (27). Socioeconomic status also plays a key role—lower socioeconomic status is associated with reduced breastfeeding rates and its shorter duration (28). However, our study offers a complementary perspective: traditional objective indicators such as income, education and employment were not significant, whereas the subjective perception of housing conditions emerged as a key predictor, the effect of which was moderated by maternal stress level.

In the context of the above findings, the present results indicate that the role of classic socio-demographic variables becomes apparent, precisely with regard to high stress. In this group, older maternal age significantly increased the odds of formula supplementation (AOR ≈ 1.81), whereas having previous children was associated with its lower odds (AOR ≈ 0.52). One possible explanation is that when experiencing stress, older first-time mothers may turn to formula supplementation as a pragmatic coping strategy to manage competing demands, while mothers with previous experience may have greater confidence and established routines that help sustain breastfeeding (29–31). Under stress, functionality and time management often take priority, and feeding choices may reflect strategies aimed at preserving limited personal resources.

In our analysis, the consistently significant role is demonstrated regarding the subjective assessment of housing conditions in both of the analyzed groups, yet with extremely different effect directions. In the group of women experiencing high stress levels, significant predictors of formula supplementation were maternal age, subjective assessment of housing conditions and parity. This means that older age increased the odds of supplementation by about 81%, whereas having children and better housing conditions both had a protective effect, reducing the odds by roughly half. The interpretation of these results can be based on the context of the Conservation of Resources theory (14), which assumes that stressful environments deplete the personal resources needed for breastfeeding. High stress likely depletes the psychological resources necessary to initiate and continue the demanding task of breastfeeding. In this situation, good, safe and satisfying housing conditions can constitute a key environmental resource, buffering the negative effects of stress, providing space for rest and recovery and thereby supporting the mother in breastfeeding (32). This is consistent with studies linking poorer housing conditions with increased parental stress and worse child health outcomes (33).

Notably, an inverse relationship was observed in the low/moderate stress group. For the model analyzed in the group of women experiencing low or moderate stress, the only significant predictor of supplementing the child with formula was the subjective assessment of housing conditions. This means that women who evaluated their housing conditions more positively were at an approximately 63% higher odds of supplementation compared to those who assessed their conditions worse. This seemingly paradoxical result may reflect that, in the low-stress group, material and infrastructural resources (e.g., financial stability, household convenience) outweigh psychological factors. Mothers judging their conditions as good may simply have greater financial and logistical capabilities to freely choose their feeding method, including resorting to formula, without the pressure induced by high stress.

Finally, the low values of the model fit indices (R^2^) deserve particular attention. They explain only 1.6% of the variability in feeding behaviors in the low/moderate stress group. This clearly indicates that socio-demographic factors, although significant, are only a small part of the multifactorial etiology of feeding behaviors. This observation is confirmed by current evidence indicating that the co-occurrence of lactation difficulties and high maternal stress levels are important warning signs (34). This suggests that the dominant factors driving feeding choices lie elsewhere—likely in psychological factors (e.g., self-efficacy, attitude), practical support (e.g., partner’s help, lactation counseling) and cultural norms (35–38).

These findings provide empirical support for the Conservation of Resources theory, demonstrating that maternal stress interacts with environmental and personal resources to shape feeding behavior. Under conditions of resource depletion, such as high stress, the availability of supportive environmental factors becomes critical for sustaining breastfeeding.

### 4.3 Public health implications

In light of the above, providing appropriate emotional and educational support, tailored to different feeding types, could be a significant protective factor (25, 26). This is also confirmed by the published research results of the current authors, which showed that among women experiencing higher stress levels, counseling on effective breastfeeding and receiving practical help were significantly associated with a reduced odds of ceasing exclusive breastfeeding (13, 16). This observation is confirmed by the latest research directions, which emphasize the importance of psychological conditions, social support (particularly from one’s partner), as well as obstetric care in shaping infant feeding decisions and practices (21, 37, 39, 40).

It is crucial to recognize that breastfeeding should not be seen as the sole responsibility of the mother; in numerous literature reviews, it is indicated that improving population-level rates requires coordinated efforts from society as a whole (18). This thesis is confirmed in our study—the fact that classic objective socio-economic variables (income, education, employment) were not significant predictors, while the subjective perception of one’s own environment was of significance, emphasizes that the mother’s life experiences and sense of security are more influential than her objective status alone. This shows how important support is when it addresses the mother’s well-being and her perceived challenges, not just material circumstances.

The empirical findings indicate a necessity to transition from universally applied approaches based solely on demographic profiles towards integrated strategies in public health interventions. Within the context of these findings, the role of midwives may be expanded to include mental health promotion through the provision of breastfeeding interventions that are also responsive to the emotional needs of mothers. Essential measures involve the reinforcement of lactation support systems, implementation of routine screening for maternal stress and associated challenges and the development of specialized, stress-reduction initiatives. Furthermore, it is fundamental to recognize breastfeeding as a collective responsibility. The formation of a comprehensive support network comprising partners, family members and healthcare professionals is critical for enhancing breastfeeding prevalence and, consequently, improving health outcomes for both mothers and infants in Poland. These recommendations are consistent with current European midwifery and public health priorities, which underline early detection of psychosocial risk factors and integrated postnatal support within primary care systems.

At an international level, the present findings align with global breastfeeding promotion strategies endorsed by the World Health Organization and UNICEF, including the *Global Nutrition Targets 2025* and the *Baby-Friendly Hospital Initiative (BFHI)* (10, 20, 41). These programs emphasize the need for comprehensive, multi-level interventions addressing not only the structural but also psychosocial determinants of breastfeeding. Our results support this approach by demonstrating that subjective factors—such as perceived security and stress—play a key role alongside socio-demographic variables. Integrating stress screening and maternal well-being support within existing breastfeeding promotion frameworks could therefore enhance the effectiveness of national and international public health initiatives.

In practice, this highlights the need to implement routine stress screening during perinatal care, integrate psychological support within lactation counseling and develop community-based programs that strengthen both emotional and practical support networks for mothers.

### 4.4 Future directions

Future studies should include longitudinal designs to clarify causal relationships between stress, lactation challenges and feeding choices. Incorporating physiological stress biomarkers (e.g., cortisol, heart-rate variability) alongside perceived stress measures could improve accuracy. Ecological momentary assessment methods might capture within-person fluctuations in stress and feeding behaviors. Furthermore, testing multicomponent interventions that integrate lactation counselling with stress-reduction strategies and resource enhancement—grounded in the Conservation of Resources framework—would provide stronger evidence for targeted public health actions.

### 4.5 Study limitations and strengths

The interpretation of the findings should consider several methodological limitations. The cross-sectional design precludes causal inference; thus, longitudinal studies are needed to better trace the temporal dynamics of stress and feeding decisions. The use of self-reported data, particularly for the assessment of maternal stress, introduces potential biases, including social desirability and recall bias. Although the PSS-10 is a validated instrument, it captures perceived rather than physiological stress, underscoring the need to include biological markers (e.g., cortisol) in future research.

The online and social media-based recruitment strategy, while allowing wide reach, may have introduced both selection and self-selection bias, as participation was more likely among mothers interested in infant feeding or maternal health topics. The sample also showed an overrepresentation of women with higher education, which may limit the generalizability of the results to the broader Polish population. Nonetheless, this reflects current demographic trends in Poland, where educational attainment among women continues to increase (42, 43). The study’s national context may further limit extrapolation of the results to settings with different healthcare systems and cultural norms. Additionally, demographic data for excluded participants (*n* = 111) were not collected, precluding assessment of possible exclusion bias. The relatively low explanatory power of the models suggests that future research should integrate a broader spectrum of psychological, cultural and environmental determinants to better capture the multifactorial nature of infant feeding practices.

While our stratified analysis by stress levels provided clear insights into context-specific mechanisms, this approach differs from testing formal interaction terms in a unified model. Future research could complement our findings by incorporating interaction analyses to formally test the statistical significance of stress as a moderating variable, while longitudinal and mixed-methods studies could further validate these findings by combining quantitative stress measures with qualitative insights into maternal decision-making.

Despite these limitations, the study has notable strengths. Its contribution lies in moving beyond analysis of simple demographic determinants and revealing the moderating role of maternal stress levels, constituting a continuation and development of the authors’ previous research in this area. The application of validated logistic regression models enabled the identification of stress-level-dependent predictors. By combining stress analysis with the subjective assessment of living conditions, the study offers a more complete and realistic image of the challenges faced by mothers. The findings are consistent with international literature on stress and lactation, while providing specific insights for creating interventions supporting maternal health, particularly during the feeding period.

## 5 Conclusion

In this study, it is highlighted that socio-demographic factors alone are not sufficient enough to explain infant feeding practices, as their influence is strongly moderated by maternal stress. The subjective perception of housing conditions emerged as a particularly important determinant, acting in opposite directions depending on stress level, which underscores the context-dependent nature of maternal decision-making. The findings suggest that interventions aimed at encouraging breastfeeding should not only address structural socio-economic inequalities but also incorporate strategies to reduce maternal stress and provide psychological support. Overall, in this study, novel evidence is provided, noting that maternal stress acts as a contextual amplifier of socio-demographic influences on feeding decisions. These insights can inform the design of targeted, resource-oriented interventions that strengthen maternal resilience and promote sustained breastfeeding at both national and European levels.

## Data Availability

The raw data supporting the conclusions of this article will be made available by the authors, without undue reservation.
