# Supplementary figures and images for: Stress and feeding choices: How do socio-demographic factors shape formula use among Polish mothers?
Source: Front Public Health. 2025 Dec 1;13:1697153. doi: 10.3389/fpubh.2025.1697153 (PMC12702739; doi:10.3389/fpubh.2025.1697153)

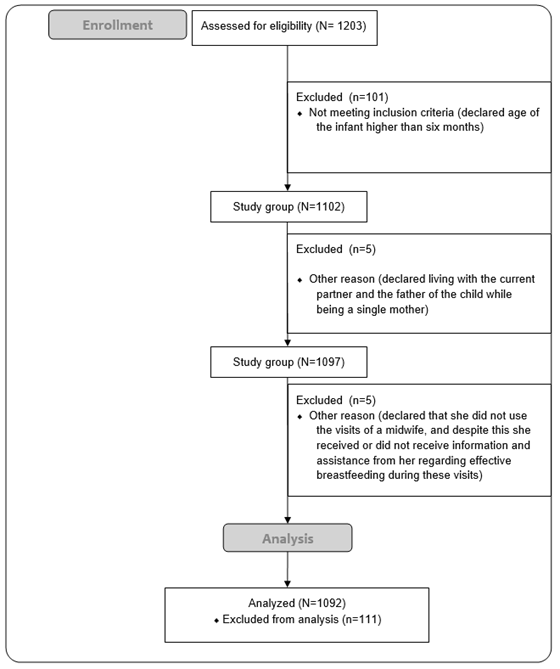

Supplement: Supplementary file 1 [file Image_1.TIF]
